# Cerebral blood volume increment after resuscitation measured by near-infrared time-resolved spectroscopy can estimate degree of hypoxic–ischemic insult in newborn piglets

**DOI:** 10.1038/s41598-021-92586-1

**Published:** 2021-06-23

**Authors:** Tsutomu Mitsuie, Shinji Nakamura, Yinmon Htun, Yasuhiro Nakao, Makoto Arioka, Kosuke Koyano, Aya Morimoto, Takayuki Wakabayashi, Yasuhiro Kuroda, Takashi Kusaka

**Affiliations:** 1 Medical Engineering Equipment Management Center, Kagawa University Hospital, Kagawa, Japan; 2 Graduate School of Medicine, Faculty of Medicine, Kagawa University, Kagawa, Japan; 3 Department of Pediatrics, Faculty of Medicine, Kagawa University, Kagawa, Japan; 4 Maternal and Perinatal Center, Kagawa University Hospital, Kagawa, Japan; 5 Department of Emergency Medicine, Faculty of Medicine, Kagawa University, Kagawa, Japan

**Keywords:** Biomarkers, Diseases, Neurology

## Abstract

Neonatal hypoxic–ischemic encephalopathy is a notable cause of neonatal death and developmental disabilities. To achieve better outcomes, it is important in treatment strategy selection to categorize the degree of hypoxia ischemia and evaluate dose response. In an asphyxia piglet model with histopathological brain injuries that we previously developed, animals survived 5 days after insult and showed changes in cerebral blood volume (CBV) that reflected the severity of injuries. However, little is known about the relationship between changes in CBV during and after insult. In this study, an HI event was induced by varying the amount and timing of inspired oxygen in 20 anesthetized piglets. CBV was measured using near-infrared time-resolved spectroscopy before, during, and 6 h after insult. Change in CBV was calculated as the difference between the peak CBV value during insult and the value at the end of insult. The decrease in CBV during insult was found to correlate with the increase in CBV within 6 h after insult. Heart rate exhibited a similar tendency to CBV, but blood pressure did not. Because the decrement in CBV was larger in severe HI, the CBV increment immediately after insult is considered useful for assessing degree of HI insult.

## Introduction

Neonatal hypoxic–ischemic encephalopathy (HIE) is a notable cause of neonatal death and developmental disabilities^1^. In meta-analyses, nearly 50% of neonates treated with therapeutic hypothermia (TH) still have major disabilities or die due to multi-organ injuries^2^. To achieve better outcomes, categorizing the degree of the hypoxia–ischemia (HI) is important for selecting suitable candidates for TH and any additional treatment strategies^3–5^. Many studies suggest that TH provides maximum neuroprotection when initiated within 6 h of birth. Therefore, it is important to recognize the changes in cerebral hemodynamics in neonates with HIE as early as possible after HI insult.

Understanding changes in cerebral hemodynamics and cerebral oxygenation status in neonatal HIE is beneficial for the prognosis of HIE, the monitoring of ongoing therapy, and the evaluation of novel therapies. In recent years, the use of near-infrared spectroscopy (NIRS) for monitoring oxygenation of the brain has provided useful insights for the management of newborns who require respiratory support, cardiovascular support, or transfusion, have HIE, or have undergone surgery^6,7^.

In neonatal HIE, HI insult affects cerebral hemodynamics and oxygenation status due to impairment in cerebral autoregulation. Impaired cerebral autoregulation results in adverse neurological outcomes^8,9^. Because assessment of vital parameters such as heart rate (HR), mean arterial blood pressure (MABP), and systemic oxygen saturation (SaO_2_) may not always reflect the extent of brain injury^10,11^, for a complete clinical picture, assessment of neonatal cerebral oxygenation and perfusion should be performed.

We previously developed a novel asphyxia piglet model with a uniform degree of histopathological brain injuries, where animals survived 5 days after insult and showed changes in cerebral blood volume (CBV), detected using near-infrared time-resolved spectroscopy (TRS)^12^. In all piglets that received HI insult, CBV increased to the peak value before decreasing to a minimum value at the time of resuscitation. These changes in CBV during insult suggested that CBV increased in a compensatory fashion under HI and that cerebral blood flow autoregulation then became impaired, resulting in decreased CBV. In further work, we observed that the decrease in CBV during insult reflected the severity of brain injuries sustained from impaired cerebral autoregulation^12,13^. Hence, we suggested that the degree of HI insult can be estimated by measuring CBV with TRS. Furthermore, when examining CBV changes not only during insult but also after insult in piglets, we found that the increase seen in CBV within 6 h after insult reflected the severity of the histological brain injuries seen at 5 days after the insult^14^. Based on these findings in piglets, we then found that the increment in CBV during the first 6 h after birth in human neonates is an indicator of poor neural prognosis thereafter^15^.

Even though it has been proved in the piglet that changes in CBV during and after HI insult reflect severity of the brain injuries sustained, little is known about the relationship between the changes in CBV during and after insult. To unravel this relationship, it would be helpful to estimate the cerebral hemodynamic response during HI insult by evaluating the cerebral hemodynamic patterns after it. We hypothesized that piglets with a greater decrease in CBV during HI insult would show a greater increase in CBV within the first 6 h after insult. The objective of this study was thus to evaluate the relationship between the CBV changes during HI insult and within 6 h of the insult in the asphyxiated piglet.

## Results

Physiological parameters are shown in Table 1. Piglets in the control group showed no significant differences versus baseline values. All parameters were compared with their respective baseline values. In the HI group, pH and base excess decreased at the time of resuscitation and had returned to baseline at 60 min after insult. Blood glucose increased at the start of resuscitation and returned to baseline 360 min after insult, whereas lactate had not returned to baseline by 360 min. PaCO_2_ and PaO_2_ values at resuscitation were 31.2 (7.4) and 16.3 (5.1) mmHg, respectively. After resuscitation, both values were within the normal range. Compared with the control group, a significant difference was seen in pH, pO_2_, BE, blood glucose, lactate, and rectal temperature immediately before resuscitation in HI piglets and pH, BE, and lactate continued in the same manner until 60 min after insult.

**Table 1:** Values of arterial gas and physiological parameters at baseline, at end of HI insult, and at 60 min, 180 min, and 360 min after insult.

	Baseline	End of insult	60 min after HI insult	180 min after HI insult	360 min after HI insult
pH	7.46 (0.07)	6.90 (0.11)****	7.32 (0.08)****	7.48 (0.07)	7.48 (0.09)
PaCO_2_ (mmHg)	41.7 (6.2)	31.2 (7.4)****	40.2 (11.3)	41.5 (9.3)	42.0 (10.9)
PaO_2_ (mmHg)	104.0 (22.2)	16.3 (5.1)****	99.2 (23.8)	89.8 (20.4)	98.1 (22.4)
Base excess (mmol/L)	5.00 (2.2)	− 25.7 (3.6)****	− 5.8 (2.6)****	6.2 (2.4)	6.6 (2.5)*
Blood glucose (mg/dL)	152.5 (39.6)	229.9 (99.8)**	220.4 (68.4)****	189.3 (64.9)*	177.5 (43.9)*
Lactate (mg/dL)	21.4 (17.9)	209.1 (29.7)****	110.9 (27.1)****	38.1 (20.0)*	32.7 (13.2)
Hemoglobin (g/dL)	9.6 (1.8)	9.9 (1.8)	9.9 (1.6)	10.0 (1.7)*	9.8 (1.8)
Rectal temperature (°C)	37.9 (0.7)	37.3 (0.8)	37.8 (0.9)	38.0 (0.6)	38.1 (0.6)

Values are shown as means (standard deviation).

*HI* hypoxic–ischemic, *pH* arterial pH, *PaCO*_*2*_ arterial PCO_2_, *PaO*_*2*_ arterial PO_2_.

*p < 0.05; **p < 0.01; ***p < 0.001; **** *p* < 0.0001 versus baseline by one-way ANOVA, a post hoc analysis with Dunnett's multiple comparison test. One piglet missed blood glucose and lactate data at 6 h after HI insult, two piglets missed rectal temperature baseline data.

The data for cerebral oxyhemoglobin (oxyHb) concentration, deoxyhemoglobin (deoxyHb) concentration, total hemoglobin (totalHb) concentration, CBV, cerebral Hb oxygen saturation (ScO_2_), MABP, and HR are shown in Table 2. During insult, CBV, totalHb, and deoxyHb increased to a maximum value and then declined, whereas oxyHb and ScO_2_ decreased and maintained lower values at the end of insult. CBV, oxyHb, and totalHb had increased again at 5 min after insult and had returned to baseline by 180 min, whereas deoxyHb decreased immediately after insult and then gradually increased. MABP decreased at the end of insult, had increased at 5 min, and had returned to baseline by 60 min after insult. HR fell during insult, gradually increased from the start of resuscitation, and had stabilized by 60 min after insult.

**Table 2:** Values of oxyHb, deoxyHb, totalHb, CBV, ScO2, MABP and HR at baseline, maximum value during insult, at the end of insult, and at 5, 60, 180, and 360 min after insult.

Parameter	Baseline	Maximum	End of HI insult	5 min after HI insult	60 min after HI insult	180 min after HI insult	360 min after HI insult
OxyHb (μM)	60.9 (10.1)	33.2 (8.3)***	34.3 (12.0)***	87.2 (13.8)***	66.4 (11.2)	60.9 (8.5)	57.9 (8.9)
DeoxyHb (μM)	21.2 (2.3)	76.8 (13.5)***	57.0 (10.8)***	12.9 (2.8)***	20.9 (4.0)	21.0 (2.7)	20.1 (3.3)
TotalHb (μM)	82.1 (10.4)	110.0 (13.3)***	91.1 (14.6)**	100.2 (13.7)***	88.1 (12.2)	81.9 (8.4)	78.0 (10.3)
CBV (mL/100 g brain tissue)	5.2 (1.0)	7.0 (1.1)***	5.8 (1.2)**	6.4 (1.1)***	5.6 (0.9)	5.1 (0.8)	5.1 (1.0)
ScO_2_ (%)	73.8 (3.8)	30.3 (7.3)***	37.2 (8.9)***	86.9 (3.2)***	76.0 (5.3)	74.1 (4.4)	74.1 (3.8)
MABP (mmHg)	76.9 (11.8)	73.7 (10.3)	44.8 (8.0)****	87.1 (12.0)*	67.6 (8.6)*	65.4 (10.5)***	63.3 (11.9)***
HR (bpm)	229 (35)	206 (27)***	139 (25)**	193 (32)	249 (29)	255 (31)	224 (34)

Values are shown as means (standard deviation).

*bpm* beats per minute, *CBV* cerebral blood volume, *deoxyHb* deoxyhemoglobin concentration, *HI* hypoxic–ischemic, *HR* heart rate, *MABP* mean arterial blood pressure, *oxyHb* oxyhemoglobin concentration, *ScO*_*2*_ cerebral Hb oxygen saturation, *totalHb* total hemoglobin concentration.

*p < 0.05; **p < 0.01; ***p < 0.001; ****p < 0.0001 versus baseline by one-way ANOVA, a post hoc analysis with Dunnett's multiple comparison test. Two piglets missed MABP and HR baseline data, one piglet missed their maximum, end of HI insult and at 5 min after HI insult data.

**Figure 1:**
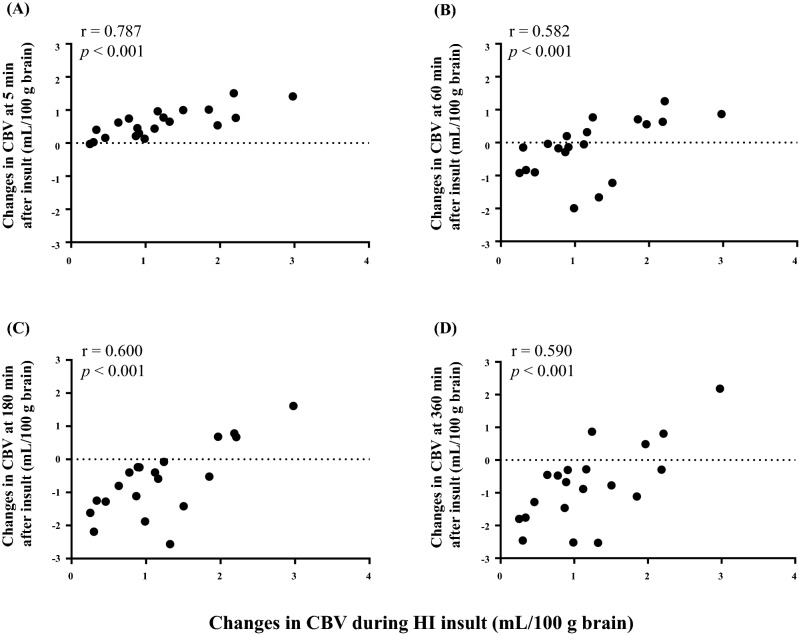
Correlation between changes in CBV during insult and changes in CBV at (**A**) 5 min, (**B**) 60 min, (**C**) 180 min, and (**D**) 360 min after insult. CBV changes were defined as the differences between the maximum CBV value during insult and the CBV values 5, 60, 180, and 360 min after insult.

**Figure 2:**
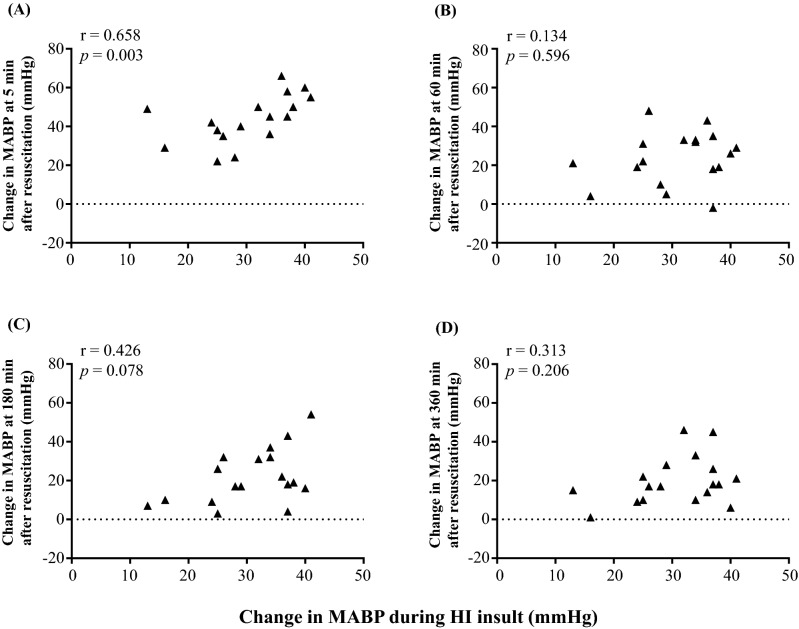
Correlation between changes in MABP during insult and changes in MABP at (**A**) 5 min, (**B**) 60 min, (**C**) 180 min, and (**D**) 360 min after insult. MABP changes were defined as the differences between the MABP value at the maximum CBV during insult and the MABP values 5, 60, 180, and 360 min after insult.

**Figure 3:**
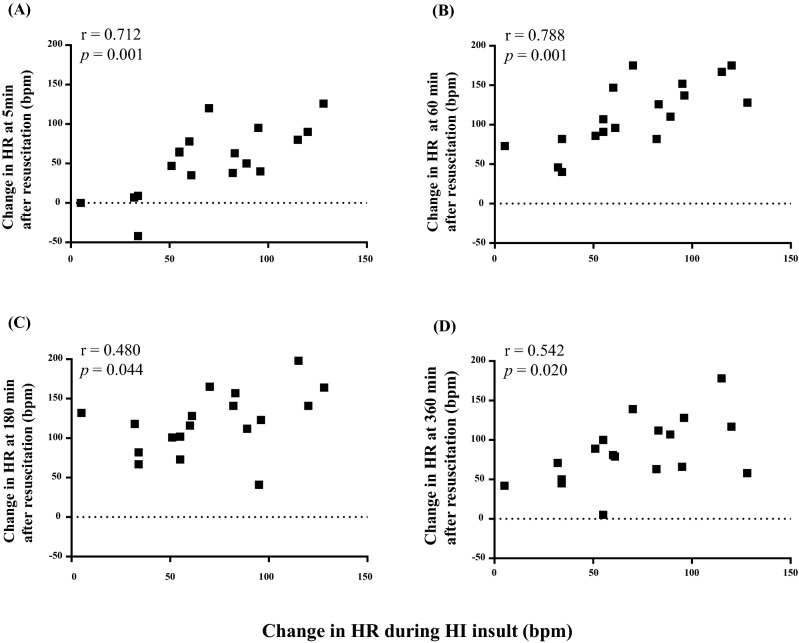
Correlation between change in HR during insult and change in HR at (**A**) 5 min, (**B**) 60 min, (**C**) 180 min, and (**D**) 360 min after insult. HR changes were defined as the differences between the HR value at peak CBV during insult and the HR value immediately before resuscitation 5, 60, 180, and 360 min after insult.

There was a positive correlation between changes in CBV during insult and changes in CBV at all time points after insult (5, 60, 180, and 360 min; Fig. 1). Similarly, the MABP increment during insult showed a positive correlation with that at 5 min after insult. However, the remaining time points showed no correlation with the MABP increment during insult (Fig. 2). The HR increment during insult and the HR increments at all time points after insult also showed a positive correlation (Fig. 3). TotalHb showed a positive correlation and ScO_2_ showed a negative correlation between changes at only 5 min and 60 min after insult, but oxyHb and deoxyHb did not show any correlations (Table 3).

**Table 3:** Correlation between changes in oxyHb, deoxyHb, total Hb and ScO2 during HI insult and at 5, 60, 180, and 360 min after insult.

	5 min after HI insult		60 min after HI insult		180 min after HI insult		360 min after HI insult	
	r	*p* value	r	*p* value	r	*p* value	r	*p* value
OxyHb	0.44	0.05	0.42	0.07	0.18	0.43	0.17	0.46
DeoxyHb	0.05	0.84	0.16	0.51	0.02	0.92	0.01	0.95
TotalHb	0.77	< 0.0001	0.68	< 0.001	0.44	0.05	0.39	0.09
ScO_2_	− 0.74	< 0.001	− 0.65	< 0.01	− 0.20	0.40	− 0.23	0.33

*deoxyHb* the concentration of deoxyhemoglobin, *HI* hypoxic–ischemic, *oxyHb* the concentration of oxyhemoglobin, *ScO*_*2*_ cerebral Hb oxygen saturation, *totalHb* the concentration of total hemoglobin.

## Discussion

In this study, we have revealed the relationships between the decrease in CBV during HI insult and the increase in CBV within 6 h of the insult in HIE piglets. This CBV decrease during insult and increase within 6 h after insult was correlated. HR showed a similar tendency to CBV but MABP did not.

During HI insult, CBV increases rapidly in a compensatory fashion, followed by impaired cerebral blood flow autoregulation and vasoparalysis that result in gradually decreased CBV due to decompensation^16,17^. In our previous translational HI piglet studies, greater decreases in CBV from baseline during insult were associated with severe brain damage or death^12^.

With respect to the increase in CBV after HI insult, we have two theories to explain why greater decreases in CBV during insult were followed by greater increases in CBV after insult. The first is severe cerebral vasoparalysis due to impaired cerebral autoregulation. Cerebral hypoperfusion, and thus decreased CBV, which are induced by severe systemic hypotension, would impair cerebral vascular autoregulation during the HI insult. After the initial resuscitation, cerebral blood flow would become passive due to a rise in systemic BP and result in an increase in CBV in the acute period immediately after resuscitation. The second is cerebral venous congestion due to heart failure, although we failed to identify a relationship between the CBV increase and the severity of the cardiac dysfunction from the data obtained in the present study, such as HR and BP. Our previous studies showed that increases in CBV at 1, 3, and 6 h after insult were associated with depressed neurocortical electrical activity at the respective time points^13^ and also histopathological brain injury at 5 days after insult^14^.

Hence, this sequence of more pronounced cerebral hypoperfusion during insult being followed by greater cerebral hyperfusion after insult reflected impaired cerebral autoregulation and resulted in severe brain injuries. In clinical practice, HIE neonates are at risk of cerebral blood flow dysregulation. Several studies have shown that impaired cerebral autoregulation (pressure-passive cerebral blood flow) after birth was associated with poor neurological outcomes^18^ and increased mortality^19^. Therefore, our work additionally suggests that CBV monitoring with TRS within the first 6 h after birth can estimate the degree of hypoperfusion during labor in HIE neonates and, further, can categorize the severity of brain injuries by recognizing the patterns of sequential changes in CBV during and after insult.

The increment in HR during insult and the increments in HR at all time points after insult showed a positive correlation. Significant cardiovascular dysfunction with redistribution of blood flow occurs in HI. In the initial stages of HI, cardiac output (CO) is well compensated and the distribution of blood to organs is maintained. However, blood is gradually redistributed to vital organs such as the brain and heart^20,21^. Myocardial ischemia results in ventricular dysfunction, which leads to a fall in stroke volume. Despite this reduced stroke volume, CO remains unchanged due to increased HR in the compensation phase, and HR also falls in the decompensation phase. The increment in HR after insult is to deliver the necessary oxygen, and the amount of increment depends on the degree of HI insult. This result is similar to that of another study in which fetal sheep were subjected to complete umbilical cord occlusion. The severe insult group showed more tachycardia than the mild insult group, and this phenomenon was related to active suppression of autonomic activity of the brain stem^22^.

MABP changes during HI constitute a complex phenomenon. MABP is influenced by multiple factors, including CO, autonomic function, neuroendocrine response, degree of vasoparalysis, and peripheral resistance^20,23^. In HI neonates, autonomic dysfunction with attenuation of parasympathetic activity and increased sympathetic activity influence the hemodynamic changes^24,25^. During HI, due to the autonomic dysfunction, compensatory tachycardia and increased BP occur initially and are followed by decompensation with a fall in HR and BP. After resuscitation, although both HR and MABP increased, MABP had returned to the level before insult earlier than HR did, and this was the reason for the lack of association between MABP increment at any time points except 5 min after insult and that during insult. This phenomenon is similar to that observed by Ikeda et al.^26^, who investigated moderate histologic damage in fetal lambs with umbilical cord ischemia. It is also similar to the findings of Yamaguchi et al.^22^ who found that 25 min of complete umbilical cord occlusion led to MABP decreasing more rapidly than HR over 6 h. On the other hand, HR and MABP were not associated with ScO_2_ and tissue perfusion index after resuscitation, indicating the difficulties of predicting cerebral circulatory disturbance^27^.

**Figure 4:**
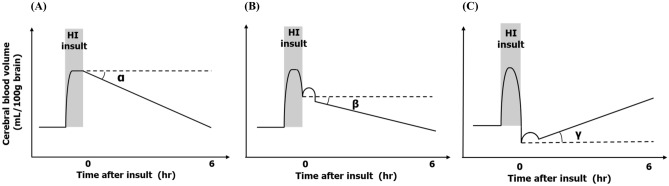
Schematic representation of the three types of changes in CBV during HI insult and after insult according to severity of insult. (**A**) In mild insults, CBV falls slightly during the insult and decreases to baseline after resuscitation. (**B**) In moderately severe insults, CBV decreases during the insult above the baseline and decreases after resuscitation to a smaller extent than in (**A**). (**C**) In severe insults, CBV falls during the insult to below the baseline and increases after resuscitation. The angles of the CBV changes from the basal horizontal line after resuscitation are α < β < γ (the angle values of α and β are negative, whereas that of γ is positive).

A graphical summary of this study and our previous work with the HI piglet model is shown in Fig. 4. In our previous study, the piglets whose CBV value was lower at the end of HI insult than at baseline showed poor prognosis, with almost all dying after insult, but almost all the piglets whose CBV value at the end of insult was higher than at baseline survived^12^. Another of our studies showed that increments in CBV after HI insult were well correlated with histopathological scores, especially at 1 and 3 h after resuscitation^14^. The results of the present study show that a greater decrease in CBV during HI insult leads to a greater increase in CBV within the first 6 h after insult, which indicates that an increase in CBV after insult can provide information not only on the degree of insult, but also on subsequent prognosis. Using these results, we can categorize three patterns of changes in CBV during and within 6 h after HI insult. A schematic representation of the patterns of changes in CBV are shown according to severity of insult: (A) in mild HI insults, a slight CBV decrease during the insult and a decrease to baseline after the insult; (B) in moderately severe insults, a CBV decrease during the insult above the baseline and a decrease after the insult that is smaller than that of (A); and (C) in severe insults, a CBV decrease during the insult to below the initial basal level of CBV and an increase after the insult. The angles of the CBV changes from the basal horizontal line after insult are α < β < γ (angle values of α and β are negative, whereas that of γ is positive). The categorization of each group was related to the prognosis within 5 days after insult. In pattern (A), the piglets all survived 5 days after the insult with no obvious neural pathological damage. In pattern (B), the piglets survived, but they had neural pathological damage. In pattern (C), the piglets did not survive after the insult due to severe convulsions or cardiac and respiratory failure. Thus, we can categorize the animals into groups by using the changes in CBV measured by TRS within 6 h after the insult to estimate future prognosis. This categorization could be applied to neonates with asphyxia to predict prognosis and we will plan to investigate this in future work.

The limitations of this study are as follows. Extra cerebral layer (skull and scalp) signals that influence the NIRS signal are not negligible in cerebral measurements. Milej et al.^28^ reported TRS measurements could reduce the contribution of the scalp to brain hemodynamic signals by an interoptode distance of over 3 cm in human adults. Because human neonates have a thin skull, superficial layer contamination is weaker than in adults^29^. Kienle et al.^30,31^ investigated analytical solutions to a two-layered optical phantom model using phase-modulated spectroscopy and demonstrated that a superficial layer 4-mm thick (or less) will have only a small influence on the accuracy of the measured optical properties of an underlying thick layer. In the piglets used in the present study, the scalp and skull layers measured postmortem were about 1.5 and 1.5 mm thick, respectively^32^, which are similar to measurements obtained in other studies^33,34^. Huber et al.^33^ and Fantini et al.^34^ concluded that in the case of a relatively thin scalp and skull layer (less than 4 mm, such as in neonatal piglets), the absolute values of Hb concentration and oxygen saturation should be representative of the brain.

Carbon dioxide is one of the most potent regulators of cerebral blood flow. In this study, PaCO_2_ was 31 mmHg during the HI insult when the ventilator was not completely stopped. This low PaCO_2_ level is different from the condition of clinical HIE where hypercapnia is observed. However, the findings of our final histopathological evaluation were similar to those of HIE, and we also observed changes in the CBV characteristic of reperfusion after ischemia. Thus, the findings of this study can serve as a reference of changes in cerebral hemodynamics in clinical HIE. Also, PaO_2_ and PaCO_2_ were maintained within the normal range during the observation period after resuscitation. Hypocapnia after resuscitation is a poor prognostic factor for HIE^35,36^, and changes in CO_2_ concentration needs to be further investigated in the future.

In HIE, the important determinants of outcomes are not only the severity of HI during insult, but also the duration and frequency of the insult, sexual dimorphism, and the presence of infection/inflammation^5,37,38^. We could only assess HI severity in this study.

## Conclusion

In this study, greater decreases in CBV during HI insult were associated with greater increases in CBV after insult. The increment in CBV immediately after resuscitation is useful for assessing the degree and timing of hypoxia, and for predicting the severity of HI insult. By using TRS, evaluating CBV changes within 6 h of HI insult has the potential to categorize the severity of the HI and enable timely and appropriate therapy to be initiated.

## Materials and methods

### Ethical approval and animal preparation

The study protocol was approved by the Animal Care and Use Committee of Kagawa University (15070-1) and in accordance with Animal Research: Reporting In Vivo Experiments (ARRIVE) guidelines. The study was carried out in compliance with the ARRIVE guidelines. All methods were carried out in accordance with relevant guidelines and regulations. Four HI piglets in our previous study were excluded from the present study due to insufficient data records^14^, and six new HI piglets were added for this new analysis. A total of 23 newborn piglets within 24 h of birth (14 males, 9 females; body weight 1560–2200 g) including 3 sham controls were anesthetized and surgically prepared.

Before the experimental procedures, the piglets were placed under a radiant warmer and their activities and alertness were briefly observed. Anesthesia was induced with 1%–2% isoflurane (Forane inhalant liquid; Abbott Co., Tokyo, Japan) in air using a facemask. Each piglet was then intubated and mechanically ventilated with an infant ventilator. The umbilical vein and artery were cannulated with a 3- or 4-Fr neonatal umbilical catheter (Atom Indwelling Feeding Tube for Infants; Atom Medical Co., Tokyo, Japan). The umbilical vein catheter was placed 5 cm from the incision for blood pressure (BP) monitoring, and the umbilical artery catheter was placed 15 cm from the incision for blood sampling. After cannulation, the piglets were anesthetized with fentanyl citrate at an initial dose of 10 µg/kg followed by continuous infusion at 5 µg/kg/h and were then paralyzed with pancuronium bromide at an initial dose of 100 µg/kg followed by continuous infusion at 100 µg/kg/h. Maintenance solution (electrolytes plus 2.7% glucose [KN3B]; Otsuka Pharmaceutical Co., Tokyo, Japan) was infused continuously at a rate of 4 mL/kg/h via the umbilical vein (glucose was infused at a rate of 2 mg/kg/min). Arterial blood samples were taken at critical points and when clinically indicated throughout the experiment. Each piglet was then placed in a copper mesh-shielded cage under a radiant warmer to maintain a rectal temperature of 38.0 ± 0.5 °C. Inspired gas was prepared by mixing O_2_ and N_2_ gases to obtain the oxygen concentrations required for the experiment. Ventilation was adjusted to maintain PaO_2_ and PaCO_2_ within their normal ranges. Arterial BPs were measured and recorded via the umbilical arterial catheter.

### Time-resolved near-infrared spectroscopy and analysis

A portable three-wavelength TRS system (TRS-10; Hamamatsu Photonics K.K., Hamamatsu, Japan) was applied using probes attached to the head of each piglet. The light emitter and detector optodes were positioned on the parietal region with a 30-mm interoptode distance. In the TRS system, a time-correlated single-photon counting technique is used for detection. The oxyHb and deoxyHb concentrations were calculated from the absorption coefficients of oxidized Hb and deoxidized Hb, under the assumption that background absorption was due only to 85% (by volume) water. TotalHb, ScO_2_, and CBV were calculated as described previously^32,39^.

### Hypoxic–ischemic insult protocol

The protocol is described in detail in our previous studies^3,12–14^. Briefly, after anesthesia induction, the piglets were stabilized. The HI insult was induced by decreasing the fraction of inspired oxygen (FiO_2_) to 4%. Low-amplitude aEEG (LAEEG < 5 μV) was achieved by additional reductions of FiO_2_ to no less than 2%. FiO_2_ was adjusted during the insult to maintain LAEEG at < 5 μV, heart rate (HR) at > 130 beats/min, and mean arterial BP (MABP) at > 70% of baseline. The insult was terminated by resuscitation with 100% FiO_2_ for 10 min. Control animals (n = 3) received 21% FiO_2_ for the duration of the experiment. CBV, vital parameters, and aEEG were measured continuously for 360 min after insult.

### Data analysis

The MABP, HR, and CBV values were analyzed from before insult to 360 min (6 h) after it. CBV changes during insult were defined as follows: Changes in CBV during insult = (maximum CBV value during insult) − (CBV value at the start of resuscitation).

CBV was measured continuously for 6 h after resuscitation. Changes in CBV values at 5, 60, 180, and 360 min after insult were calculated as follows: Changes in CBV at each time point = (CBV values at each time point) − (CBV value at the start of resuscitation).

**Figure 5:**
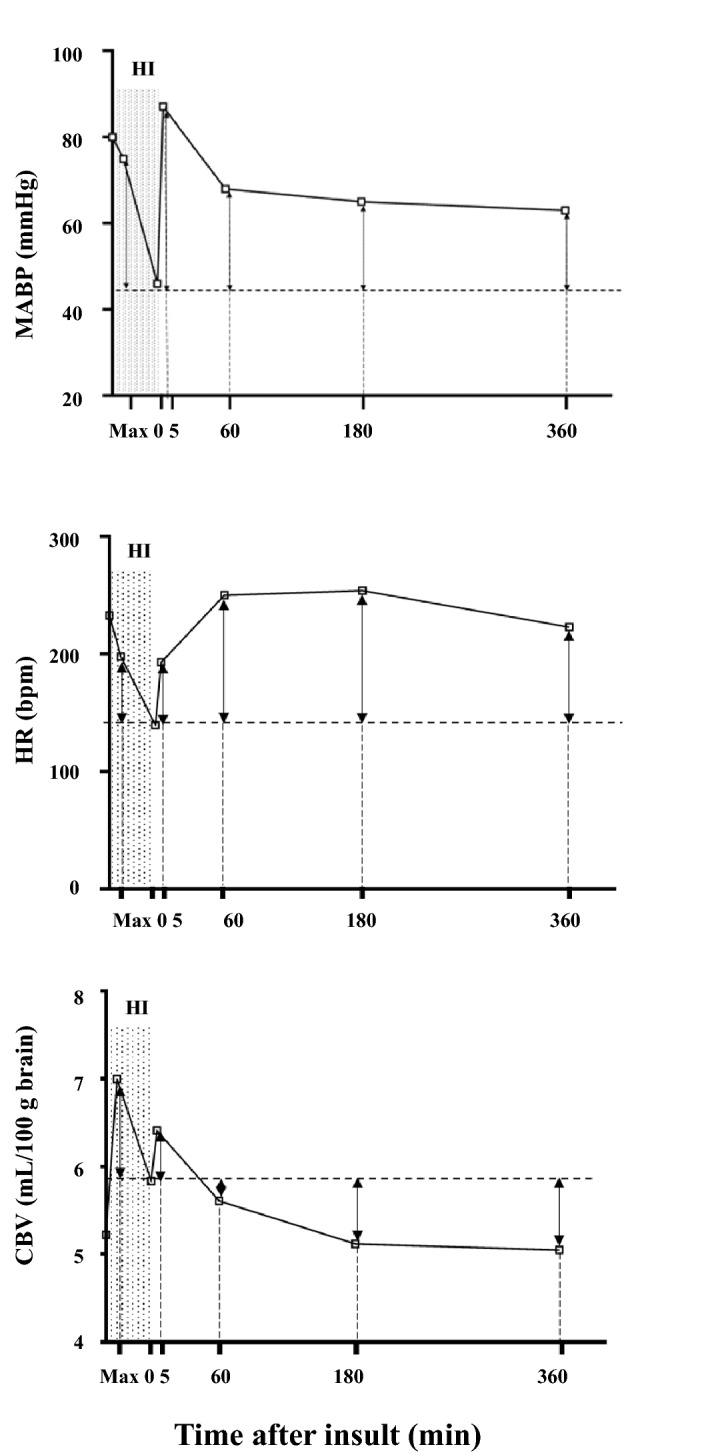
Graphical representation of the increments in CBV, HR, and MABP during HI insult and at 5, 60, 180, and 360 min after insult. Increments in CBV were calculated as the differences between the CBV value at the end of insult and the maximum CBV value during insult and the CBV at 5 min, 60 min, 180 min, and 360 min after insult. Increments in HR were calculated as the differences between the HR value at the end of insult and the HR value at the maximum CBV during insult and the HR at 5 min, 60 min, 180 min, and 360 min after insult. Increments in MABP were calculated as the differences between the MABP value at the end of insult and the MABP value at the maximum CBV during insult and the MABP at 5 min, 60 min, 180 min, and 360 min after insult.

In addition, the following relationships were analyzed (Fig. 5): (1) relationship between changes in CBV during insult and changes in CBV at each time point after insult; (2) relationship between the MABP increment at maximum CBV during insult and MABP increment at each time point after insult; and (3) relationship between the HR increment at maximum CBV during insult and HR increment at each time point after insult.

### Statistical analysis

GraphPad Prism 5J (GraphPad Software, La Jolla, CA) was used for all statistical analyses. Significant correlations were assessed by Spearman’s p rank test for the relationship between changes in CBV, HR, and MABP during and after insult. Physiological variables of the HI group were compared with those of the control group using the Mann–Whitney *U* test and, in each group, these variables were compared with those of pre-baseline data using Dunnett's multiple comparison test. Statistical significance for all tests was set at *p* < 0.05. All values are presented as means ± SD.
